# Blast Alleviation of Sacrificial Cladding with Graded and Uniform Cellular Materials

**DOI:** 10.3390/ma13245616

**Published:** 2020-12-09

**Authors:** Yuanyuan Ding, Yuxuan Zheng, Zhijun Zheng, Yonggang Wang, Siyuan He, Fenghua Zhou

**Affiliations:** 1 Key Laboratory of Impact and Safety Engineering, Ministry of Education, Ningbo University, Ningbo 315211, China; zhengyuxuan@nbu.edu.cn (Y.Z.); wangyonggang@nbu.edu.cn (Y.W.); zhoufenghua@nbu.edu.cn (F.Z.); 2 CAS Key Laboratory of Mechanical Behavior and Design of Materials, Department of Modern Mechanics, University of Science and Technology of China, Hefei 230026, China; 3 State Key Laboratory of Bioelectronics, Southeast University, Nanjing 210096, China; siyuan_he@seu.edu.cn

**Keywords:** cellular sacrificial cladding, density distribution design, shock wave, analytical model, blast alleviation

## Abstract

Graded cellular material is a superb sandwich candidate for blast alleviation, but it has a disadvantage for the anti-blast design of sacrificial cladding, i.e., the supporting stress for the graded cellular material cannot maintain a constant level. Thus, a density graded-uniform cellular sacrificial cladding was developed, and its anti-blast response was investigated theoretically and numerically. One-dimensional nonlinear plastic shock models were proposed to analyze wave propagation in density graded-uniform cellular claddings under blast loading. There are two shock fronts in a positively graded-uniform cladding; while there are three shock fronts in a negatively graded-uniform cladding. Response features of density graded-uniform claddings were analyzed, and then a comparison with the cladding based on the uniform cellular material was carried out. Results showed that the cladding with uniform cellular materials is a good choice for the optimal mass design, while the density graded-uniform cladding is more advantageous from the perspective of the critical length design indicator. A partition diagram for the optimal length of sacrificial claddings under a defined blast loading was proposed for engineering design. Finally, cell-based finite element models were applied to verify the anti-blast response results of density graded-uniform claddings.

## 1. Introduction

Cellular materials have been popularly regarded as an ideal anti-blast/cushion material due to their superior energy absorption capability and shock mitigation [1,2,3,4]. The primary applications of cellular materials are energy protectors (such as helmets and shields) and packaging of fragile components. Under blast/impact loading, cellular materials can undergo a large deformation and simultaneously dissipate shock energy with a low transmitted stress. Thus, sandwich structures with cellular materials as core materials appear to be gradually within our sight. Cellular sacrificial cladding, a kind of sandwich structure, is composed of two cover plates and a cellular material core, where the function of the cover plates is to improve the flexural rigidity of sacrificial cladding and to avoid nonuniform blast loading. When subjected to impact/blast loading, cellular sacrificial cladding can absorb enormous energy with a layer-wise deformation mode and can protect the main structure behind the cladding from destruction [5,6].

It is a crucial point to understand the mechanical behavior of cellular material for guiding the anti-blast design of cellular sacrificial cladding. Under impact/blast loading, cellular material deforms with two typical features, namely deformation localization and stress enhancement, which can be well characterized by shock models [7,8,9,10,11,12,13,14,15]. A shock model was first proposed by Reid and Peng [7] to describe the dynamic behavior of the wood based on a rate-independent, rigid-perfectly plastic-locking (R-PP-L) idealization, and it has been subsequently applied to many other porous materials [8,16,17,18], such as honeycomb [16], metal lattice [17] and aluminum foam [8,18]. Recently, Zheng et al. [12] explored the deformation mechanism related to the nonlinear plastic hardening effect of cellular materials and proposed a rate-independent, rigid-plastic hardening (R-PH) idealization to characterize the quasi-static compression stress-strain behavior. The R-PH material model can well fit the nominal stress-strain curve of cellular materials. Ding et al. [2] further demonstrated that the R-PH model is more precise than the R-PP-L model to evaluate the anti-blast properties of the sacrificial cladding with uniform cellular material.

In the structural design of cellular sacrificial cladding, researchers gradually looked forward to graded cellular materials due to their designable properties. Ma and Ye [19] proposed a double-layer foam cladding with different densities, and the further study of Liao et al. [20] showed that the double-layer sacrificial cladding was more efficient than the single-layer sacrificial cladding. Karagiozova and Alves [21] analyzed the wave propagation in layered cellular solids under impact loading, and their results suggested that the weakest material should be placed near the stationary end to ensure a smaller stress transmitted to the main structure. Recently, the dynamic response of continuous graded cellular materials was investigated and the results illustrate that their dynamic energy absorption can be improved [22,23,24,25,26,27,28,29], compared to the homogenous rod. The anti-blast behavior of graded cellular sacrificial cladding is studied in the literature [30,31,32,33,34], which brought some beneficial understandings, e.g., the thickness of the graded cladding needing to absorb the blast loading is smaller with a more significant density gradient parameter. However, the supporting stress that the stress at the supporting end of the structure, for graded cellular claddings (including both the positive and negative density distribution cases) under dynamic loading cannot maintain a constant level and is greatly affected by the density arrangement and dynamic loading intensity. It is disadvantageous for the blast alleviation design of graded cellular materials. Therefore, a neotype double-layer cellular cladding composed of a graded cellular layer and a uniform cellular layer, where the uniform foam layer maintains a constant transmitted stress suffered by the main structure and the graded foam layer increases the energy absorption properties, is proposed in this paper and is applied as an anti-blast structure. Thus, it can give full play to the functions of graded and uniform layers to ensure its blast alleviation.

In this paper, the anti-blast responses of density graded-uniform sacrificial cellular claddings are investigated with shock models based on the rate-independent R-PH idealization. The outline is structured as follows. The problem of density graded-uniform cellular cladding suffered by blast loading is described in Section 2. Then, in Section 3, the governing equation is derived to investigate the shock front motion in the density graded-uniform cellular cladding. The main results and discussion about the anti-blast responses of density graded-uniform cellular claddings are given in Section 4. A comparison between the cell-based finite element (FE) results and the theoretical prediction is performed in Section 5, followed by conclusions in Section 6.

## 2. Theoretical and Numerical Models

### 2.1. Problem Description

**Figure 1:**
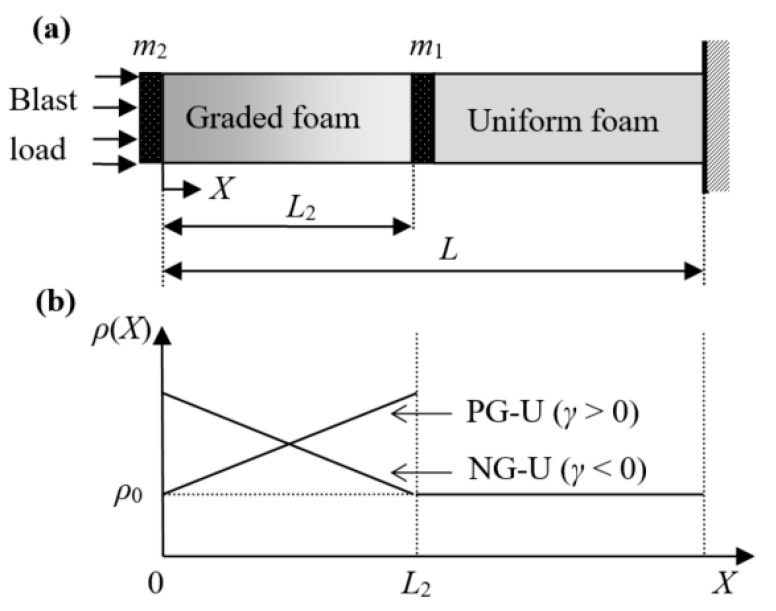
(**a**) Sketch of density graded-uniform cellular cladding for blast alleviation and (**b**) its density distribution.

We consider a new double-layer cellular cladding, namely graded-uniform cellular sacrificial cladding, as the impact alleviation, as shown in Figure 1a. According to the density distribution of the graded cellular layer, the graded-uniform cellular sacrificial cladding can be distinguished into two cases: positively and negatively graded-uniform cellular sacrificial claddings (called “PG-U cladding” and “NG-U cladding” for short, respectively). In this study, we only consider the linear density distribution of the graded cellular cladding, and the lowest density of the graded foam is equal to the density of the uniform foam. Thus, the density distribution of the graded-uniform cellular sacrificial cladding can be expressed as

(1) $$\rho_{f}\left(X\right)=\rho \left(X\right)\rho_{S}=\left\{ \begin{array}{ll} \frac{2\rho_{0}\rho_{S}}{2-\left|\gamma \right|}\left[1+\gamma \left(X/L_{2}-1/2\right)\right], & 0\leq X\leq L_{2}\\ \rho_{0}\rho_{S}, & L_{2}<X\leq L \end{array}\right.$$

where *X* is the Lagrangian coordinate, *L*_2_ is the length of the graded layer, *L* is the total length of the graded-uniform sacrificial cladding, *ρ*(*X*) represents the relative density distribution, *ρ*_0_ is the relative density of uniform cellular cladding, *γ* = 2(*ρ*(*L*_2_) − *ρ*(0))/(*ρ*(*L*_2_) + *ρ*(0)) is the density-gradient parameter and *ρ*_s_ is the density of the base material. For the case of *γ* > 0, the density increases linearly along the graded foam rod with a slope of 2*ρ*_s_*ρ*_0_*γ*/(2*L* − |*γ*|*L*_2_), and for the case of *γ* < 0, the density decreases linearly with the same slope. Two cover plates added to the cladding are used to increase the flexural capacity of the cladding, and the mass per unit area of the plates are *m*_2_ and *m*_1_. The thicknesses of both cover plates are negligible in the theoretical analysis.

The blast loading can be approximated as a plane pressure wave when the explosion takes place at a certain distance away from the cladding. In general, the blast pressure can be assumed to be an exponential attenuation wave [32,33,35,36], written as

(2) $$p(t)=P_{0}e^{-t/\tau }$$

where *t* is the time, *P*_0_ is the initial peak of the blast loading and *τ* is the decay time of the blast loading. The impulse of the blast can be obtained as *P*_0_*τ*. It should be noted that the blast loading considered here takes into account the fluid-structure interaction effects, which are the interactions between the blast pressure produced by the explosion and the motion of the cover plate of the cellular sacrificial cladding.

### 2.2. Stress-Strain Relations of Cellular Materials

**Figure 2:**
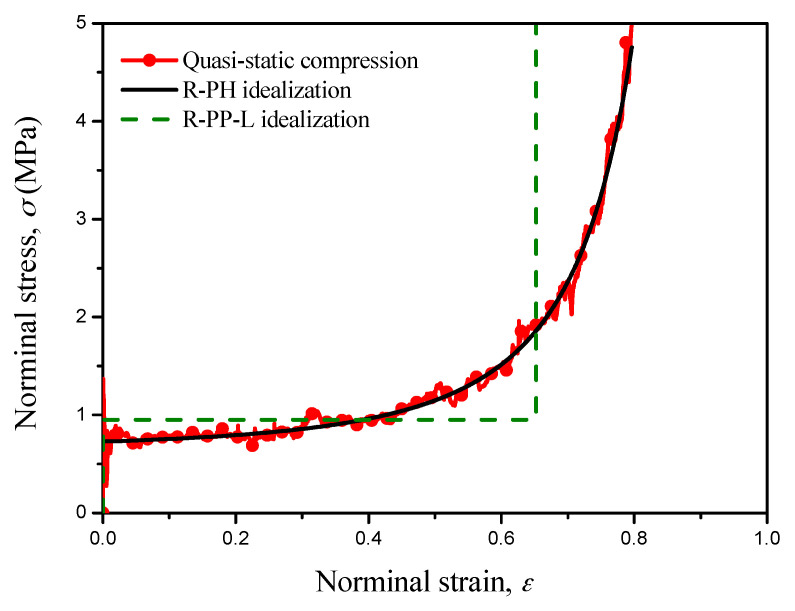
The quasi-static nominal stress strain curve obtained from the cell-based finite element (FE) model [31] and the R-PP-L and R-PH idealizations.

Due to the difficulty of obtaining the dynamic stress-strain relations of cellular materials, the quasi-static stress–strain curve is often used to evaluate and design the cellular cladding for engineering projects. The stress-strain curve of cellular materials under quasi-static compression presents three distinct regions, namely an elastic region, a long plateau plastic region and a densification region. Several simplified idealizations were therefore proposed to characterize the collapse behavior of cellular materials. The R-PP-L idealization [7,8] is the most popular one, and it only contains two material parameters, i.e., plateau stress and locking strain. However, it is demonstrated that the R-PP-L idealization can only provide a first-order approximation for the deformation behavior of cellular materials owing to the constant locking strain assumption [2]. The densification region of the quasi-static stress-strain curve presents a strongly nonlinear plastic hardening phenomenon [12,37], which may be more directly reveal the unreasonableness of the R-PP-L idealization for cellular materials. Here, the rate-independent R-PH idealization proposed by Zheng [12] is implemented to characterize and predict the energy absorption of cellular materials, as shown in Figure 2. The R-PH idealization can depict the stress-strain curves well, and it can be given by

(3) $$\sigma (\varepsilon )=\sigma_{0}(\rho )+\frac{C(\rho )\varepsilon }{{\left(1-\varepsilon \right)}^{2}}$$

where *σ*_0_(*ρ*) and *C*(*ρ*) are the initial crushing stress and the strain hardening parameter, respectively, which are related to the local relative density *ρ*(*X*).

## 3. Blast Alleviation of Graded-Uniform Cellular Sacrificial Claddings

In the case of a material subjected to an increasing velocity from zero, if the material has an upward concave stress–strain relation, a series of plastic waves initially propagate from the impact end, and transform into a shock wave later when the loading velocity is high enough [38]. According to the experience, the material at the blast end of a cellular sacrificial cladding could reach a high speed in a very short time. To pursue the authenticity of the material models and blast loading [6,23], we assume that the shock wave can be formed as soon as an increasing velocity from zero acts on the cellular sacrificial cladding. The rationality of this hypothesis will be illustrated by the cell-based finite element results in Section 5.

According to the R-PH model, the elastic wave speed is infinite, and the shock wave speed is finite and determined by the slope of Rayleigh line [8]. It indicates that the shock wave cannot catch up with the elastic wave, and the stress ahead of the shock front is equal to *σ*_0_(*ρ*). Thus, the physical quantities ahead of the shock front can be expressed as {*v*_A_, *ε*_A_, *σ*_A_} = {*v*, 0, *σ*_0_(*ρ*)}. If the velocity ahead of the shock front is determined, the physical quantities behind the shock front {*v*_B_, *ε*_B_, *σ*_B_} can be obtained with the aid of Equation (3) and the conservation relations of mass and momentum across the shock front [39], which are

(4) $$\left\{ \begin{array}{l} v_{B}(t)=v_{A}(t)+v_{\mathrm{Sh}}(\varepsilon_{B}(t)-\varepsilon_{A}(t))\\ \sigma_{B}(t)=\sigma_{A}(t)+\rho \rho_{S}v_{\mathrm{Sh}}(v_{B}(t)-v_{A}(t)) \end{array}\right.$$

where *v*_Sh_ is the shock front speed and *ρ* is the local relative density in cellular material at the shock front position.

Several kinds of sacrificial claddings, serving as a protective function from blast or impact loads, were proposed and investigated theoretically, experimentally and numerically in the literature [5,6,19,20,21,22,23,24,25,26,27,28,29,30,31,32,33,34], such as single, double, triple and graded cellular sacrificial claddings. However, deformation patterns of those sacrificial claddings under impact loading are different and highly dependent on the density distribution of sacrificial cladding. Under impact loading, the single, uniform cellular layer deforms layer-by-layer, and only one shock wave can be found propagating from the proximal end to the distal end. To enhance the blast/impact resistant capacity, double and triple cellular claddings [21] are proposed, and double shock waves and triple shock waves can be found respectively under impact loading. The deformation pattern of the graded cellular layer is more complicated [22,24,25,40], and a variety of forms of shock wave propagation are possible according to the density distribution in the graded cladding. Therefore, figuring out the deformation mode of the sacrificial layer is the top priority of the anti-blast analysis of sacrificial cladding.

### 3.1. Positively Graded-Uniform Cellular Sacrificial Cladding (PG-U Cladding)

**Figure 3:**
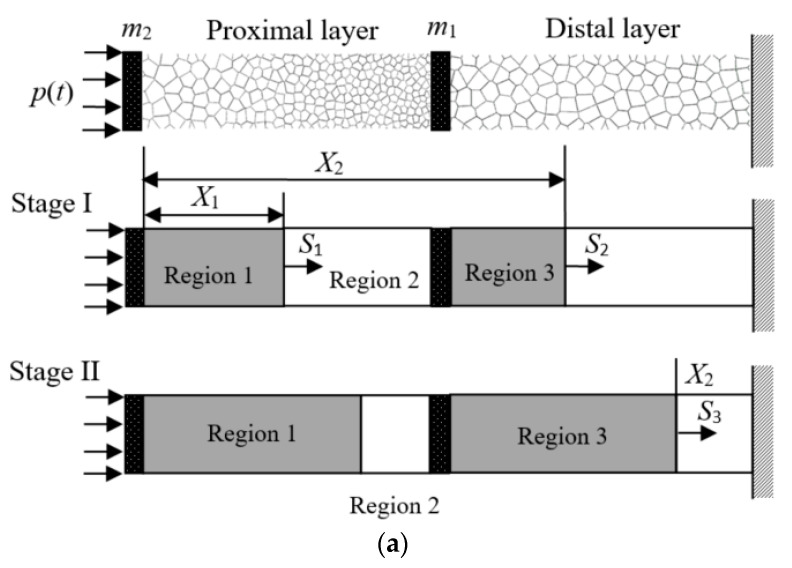

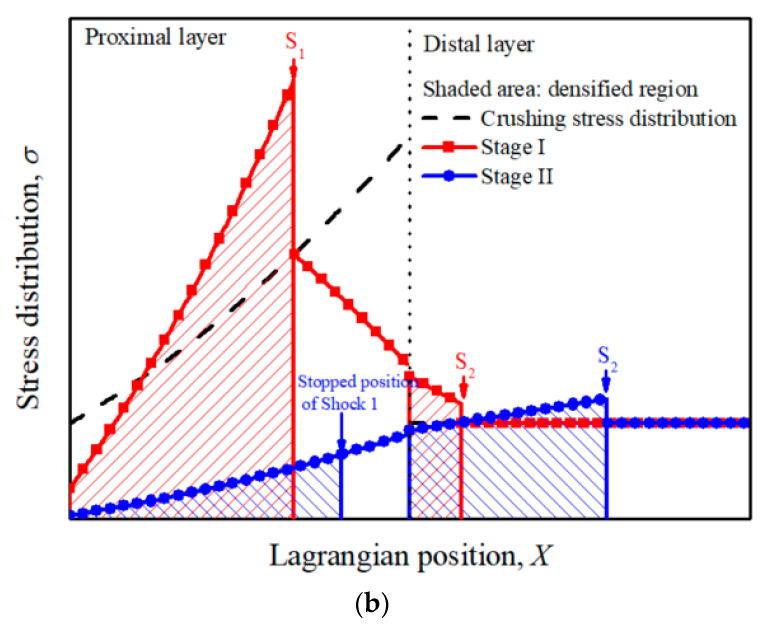
(**a**) The deformation diagram and (**b**) the stress distributions of PG-U cladding subjected to blast loading.

Consider a unit strip of a sacrificial cladding comprising a positively graded cellular layer and a uniform cellular layer, as shown in Figure 3. Once a blast loading is applied to the PG-U cladding, a shock wave initiates at the proximal end and travels through the cladding with a finite speed. If there is no other shock wave, stress imbalance would appear at the distal layer. Thus, it is assumed that two plastic shock waves simultaneously initiate in a PG-U cladding, i.e., one traveling in the proximal layer and the other propagating in the distal layer, then the proximal layer and the distal layer deform simultaneously. Before the shock wave arrives at the end of the distal layer, the stress at the support end (stress transmitted to the protected structure) is limited to *σ*_0_(*ρ*_0_), which can be set as the allowable stress of the protective structure from damage, and it can be roughly achieved when the distal layer is sufficiently thick.

Assuming the cover plate is a rigid body and the origin of the *X* coordinate is at the proximal end, as shown in Figure 3a. *X*_1_ and *X*_2_ are used to denote the Lagrangian coordinates of Shock 1 and Shock 2. Thus, the velocities of the two shock fronts *S*_1_ and *S*_2_ can be written as

(5) $$S_{1}=\frac{dX_{1}}{dt},\;S_{2}=\frac{dX_{2}}{dt}$$

According to Equation (4), the mass and momentum conservation conditions across Shocks 1 and 2 are

(6) $$\left\{ \begin{array}{l} v_{1}-v_{2}=S_{1}\varepsilon_{1}\\ \sigma_{1}-\sigma_{0}(\rho (X_{1}))=\rho_{S}\rho (X_{1})S_{1}(v_{1}-v_{2}) \end{array}\right.$$

and

(7) $$\left\{ \begin{array}{l} v_{2}=S_{2}\varepsilon_{2}\\ \sigma_{2}-\sigma_{0}(\rho (X_{2}))=\rho_{S}\rho (X_{2})S_{2}v_{2} \end{array}\right.$$

where {*v*_1_, *ε*_1_, *σ*_1_} represent the velocity, strain and stress behind Shock 1, respectively, and {*v*_2_, *ε*_2_, *σ*_2_} are those behind Shock 2, respectively.

The momentum conservation of the cover plate *m*_2_ and Region 1 at time *t* gives

(8) $$p(t)-\sigma_{1}=\left(m_{2}+\rho_{S}\int_{0}^{X_{1}}\rho \left(x\right)dx\right)\frac{dv_{1}}{dt}$$

and the similar momentum conservation of Region 2, middle plate *m*_1_ and Region 3 through time interval d*t* leads to

(9) $$\sigma_{0}(\rho (X_{1}))-\sigma_{2}=\left(m_{1}+\rho_{S}\int_{X_{1}}^{L_{2}}\rho \left(x\right)dx+\rho_{S}\rho_{0}(X_{2}-L_{2})\right)\frac{dv_{2}}{dt}$$

Combining Equations (3) and (5)–(9), we have

(10) $$\left\{ \begin{array}{l} \frac{dX_{1}}{dt}=v_{1}-v_{2}+c_{1}\\ \frac{dX_{2}}{dt}=v_{2}+c_{2} \end{array}\right.$$

and

(11) $$\left\{ \begin{array}{l} \frac{dv_{1}}{dt}=\frac{p(t)-\sigma_{0}(\rho (X_{1}))-\rho_{S}\rho (X_{1})\left(v_{1}-v_{2}\right)\left(v_{1}-v_{2}+c_{1}\right)}{m_{2}+\rho_{S}\int_{0}^{X_{1}}\rho \left(x\right)dx}\\ \frac{dv_{2}}{dt}=\frac{\sigma_{0}(\rho (X_{1}))-\sigma_{0}(\rho (X_{2}))-\rho_{S}\rho (X_{2})v_{2}\left(v_{2}+c_{2}\right)}{\left(m_{1}+\rho_{S}\int_{X_{1}}^{L_{2}}\rho (x)dx+\rho_{S}\rho_{0}(X_{2}-L_{2})\right)} \end{array}\right.$$

where $c_{1}=\sqrt{C(\rho (X_{1}))/\rho_{S}\rho (X_{1})}$ and $c_{2}=\sqrt{C(\rho (X_{2}))/\rho_{S}\rho (X_{2})}$.

Thus, the governing equations of the anti-blast response of PG-U cladding at Stage I are controlled by Equations (10) and (11). However, there is no explicit solution to those differential equations. In this study, they are solved numerically with a fourth-order Runge-Kutta scheme with the initial conditions *X*_1_ = 0, *X*_2_ = *L*_2_ and *v*_1_ = *v*_2_ = 0.

At Stage I, the velocity of Region 1 increases sharply and then decreases slowly due to the resistance of the rod for the force competition of blast loading *p*(*t*) and stress behind Shock 1, *σ*_1_. The common velocity *v*_2_ of Regions 2 and 3 is increasing due to the difference of the stress ahead of Shock 1 *σ*_0_(*ρ*(*X*_1_)) and the stress behind Shock 2, *σ*_2_. When *v*_1_ = *v*_2_, Shock 1 vanishes while Region 1, Region 2 and Region 3 with the cover plates *m*_1_ and *m*_2_ move as an entirety towards the distal end. At this moment, Stage II commences. With the application of the mass and momentum conservation relations across Shock 2, Equations (10) and (11) can be rewritten as

(12) $$\frac{dX_{2}}{dt}=v_{2}+c_{2}$$

and

(13) $$\frac{dv_{2}}{dt}=\frac{p(t)-\sigma_{0}(\rho_{0})-\rho_{S}\rho_{0}v_{2}(v_{2}+c_{2})}{m_{1}+m_{2}+2\rho_{S}\rho_{0}L_{2}/(2-\gamma )+\rho_{S}\rho_{0}(X_{2}-L_{2})}$$

The deformation at Stage II is governed by Equations (12) and (13), and the initial conditions are *X*_1_ = *x*_11_, *X*_2_ = *x*_12_, *v*_1_ = *v*_2_ = *v*_11_ = *v*_12_, where *x*_11_, *x*_12_, *v*_11_ and *v*_12_ are the Lagrangian coordinates of Shocks 1 and 2, the velocities of Regions 1 and 2 at the end of Stage I, respectively. The typical stress distribution along the cladding at Stage I and II are depicted in Figure 3b. It is obvious that the stresses at the undeformed regions do not exceed the local initial crushing stress, which illustrates that the assumption of double shock waves is self-consistent.

### 3.2. Negatively Graded-Uniform Cellular Sacrificial Cladding (NG-U Cladding)

**Figure 4:**
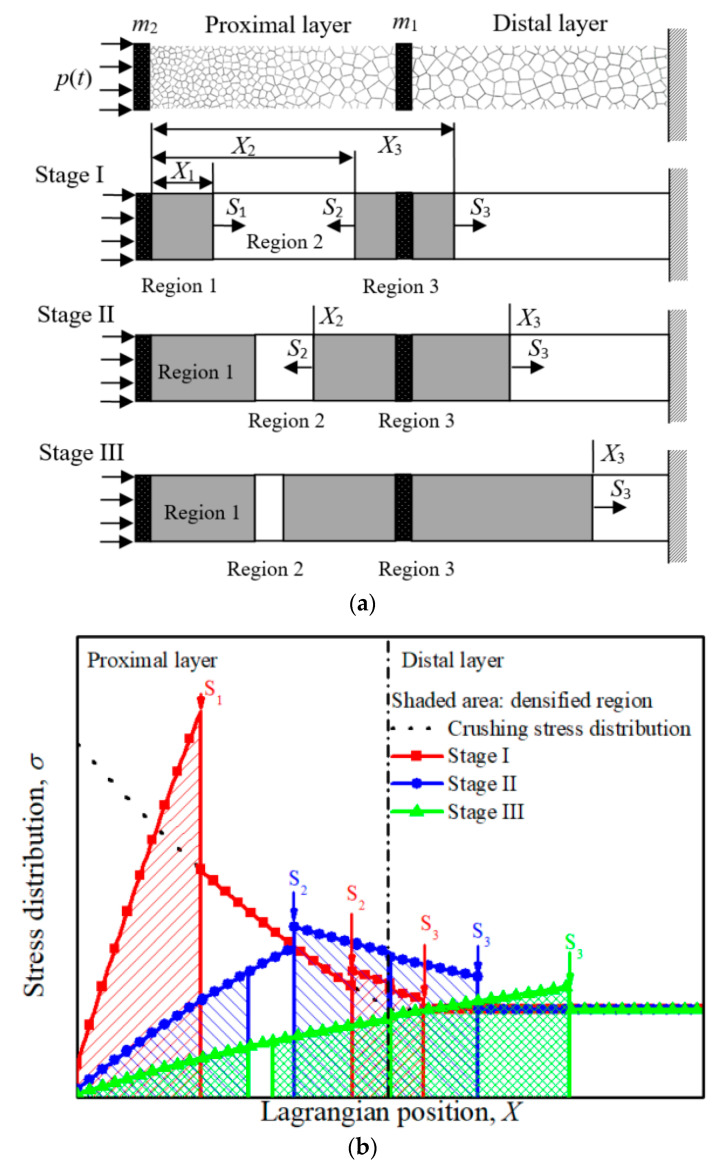
(**a**) The deformation diagram and (**b**) the stress distributions of NG-U cladding subjected to blast loading.

It is supposed that there exist three shock fronts simultaneously propagating in the NG-U cladding at Stage I, as shown in Figure 4a. Under blast loading, Shock 1 propagates from the proximal end to the distal end, while Shock 2 and Shock 3 initiate at the interface between the graded layer and uniform layer, i.e., they are the same disturbance contributed to the crushing at the weakest point. Then Shock 2 propagates towards the proximal end, and Shock 3 propagates oppositely towards the distal end. This assumption will be validated by the FE simulations in Section 5. In the process of propagation, the compacted part of Region 1 and cover plate *m*_2_ move forward with a velocity of *v*_1_, Region 2 moves with velocity *v*_2_ and Region 3 with *v*_3_. These velocities satisfy the relationship numerically: *v*_1_ > *v*_2_ > *v*_3_ at stage I.

Similar to the analysis in Section 3.1, the shock front velocities of *S*_1_, *S*_2_, and *S*_3_ are

(14) $$S_{1}=\frac{dX_{1}}{dt},S_{2}=\frac{dX_{2}}{dt},S_{3}=\frac{dX_{3}}{dt}$$

where *X*_1_, *X*_2_ and *X*_3_ are the shock position measured from the proximal end of cladding. Considering the fact that Shock 1 and Shock 3 are right-spreading waves and Shock 2 is the left-spreading wave, the conservation conditions across Shocks 1, 2 and 3 take the forms:

(15) $$\left\{ \begin{array}{l} v_{1}-v_{2}=S_{1}\varepsilon_{1}\\ \sigma_{1}-\sigma_{0}(\rho (X_{1}))=\rho_{S}\rho (X_{1})S_{1}(v_{1}-v_{2}) \end{array}\right.$$

(16) $$\left\{ \begin{array}{l} v_{2}-v_{3}=-S_{2}\varepsilon_{2}\\ \sigma_{2}-\sigma_{0}(\rho (X_{2}))=-\rho_{S}\rho (X_{2})S_{2}(v_{2}-v_{3}) \end{array}\right.$$

and

(17) $$\left\{ \begin{array}{l} v_{3}=S_{3}\varepsilon_{3}\\ \sigma_{3}-\sigma_{0}(\rho_{0})=\rho_{S}\rho_{0}S_{3}v_{3} \end{array}\right.$$

where {*v*_1_, *ε*_1_, *σ*_1_}, {*v*_2_, *ε*_2_, *σ*_2_}, {*v*_3_, *ε*_3_, *σ*_3_} represent the velocity, strain and stress behind Shocks 1, 2 and 3, respectively. Further application of the momentum conservation relations of compacted Region 1 and m2, Region 2 and Region 3, respectively, we have

(18) $$p(t)-\sigma_{1}=\left(m_{2}+\rho_{S}\int_{0}^{X_{1}}\rho \left(x\right)dx\right)\frac{dv_{1}}{dt}$$

(19) $$\sigma_{0}(\rho (X_{1}))-\sigma_{0}(\rho (X_{2}))=\rho_{S}\int_{X_{1}}^{X_{2}}\rho \left(x\right)dx\frac{dv_{2}}{dt}$$

and

(20) $$\sigma_{2}-\sigma_{3}=\left(m_{1}+\rho_{S}\int_{X_{2}}^{L_{2}}\rho \left(x\right)dx+\rho_{S}\rho_{0}(X_{3}-L_{2})\right)\frac{dv_{3}}{dt}$$

Therefore, substituting Equations (3), (15)–(17) into Equation (14) yields

(21) $$\left\{ \begin{array}{l} \frac{dX_{1}}{dt}=v_{1}-v_{2}+c_{1}\\ \frac{dX_{2}}{dt}=-(v_{2}-v_{3}+c_{2})\\ \frac{dX_{3}}{dt}=v_{3}+c_{3} \end{array}\right.$$

where $c_{3}=\sqrt{C(\rho (X_{3}))/\rho_{S}\rho (X_{3})}$. Substituting Equations (3), (15)–(17) into Equations (18)–(20) yields

(22) $$\left\{ \begin{array}{l} \frac{dv_{1}}{dt}=\frac{p(t)-\sigma_{0}(\rho \left(X_{1}\right))-\rho_{S}\rho \left(X_{1}\right)(v_{1}-v_{2})(v_{1}-v_{2}+c_{1})}{m_{2}+\rho_{S}\int_{0}^{X_{1}}\rho \left(x\right)dx}\\ \frac{dv_{2}}{dt}=\frac{\sigma_{0}(\rho (X_{1}))-\sigma_{0}(\rho (X_{2}))}{\rho_{S}\int_{X_{1}}^{X_{2}}\rho \left(x\right)dx}\\ \frac{dv_{3}}{dt}=\frac{\sigma_{0}(\rho \left(X_{2}\right))+\rho_{S}\rho \left(X_{2}\right)(v_{2}-v_{3})(v_{2}-v_{3}+c_{2})-\sigma_{0}(\rho_{0})-\rho_{S}\rho_{0}v_{3}(v_{3}+c_{3})}{m_{1}+\rho_{S}\int_{X_{2}}^{L_{2}}\rho \left(x\right)dx+\rho_{S}\rho_{0}(X_{3}-L_{2})} \end{array}\right.$$

Thus Equations (21) and (22) are the governing equations of NG-U cladding at Stage I, and its initial conditions are *X*_1_ = 0, *X*_2_ = *X*_3_ = *L*_2_ and *v*_1_ = *v*_2_ = *v*_3_ = 0. It is noteworthy that the acceleration of Region 2, d*v*_2_/d*t*, is a constant determined by the density-gradient parameter *γ* in graded layer. Thus an initial constraint d*v*_1_/d*t*|*_t_*_= 0_ > d*v*_2_/d*t*|*_t_*_= 0_ (i.e., (*P*_0_ − *σ*(*ρ*(0)))/*m*_2_ > − *4γk_1_σ_S_ρ_0_/(2+γ)L_2_ρ_S_* when using Equation (27)) should be satisfied to guarantee the shock propagation assumption in NG-U cladding. When speed *v*_1_ drops to equal *v*_2_, Shock 1 vanishes, and Shock 2 remains to propagate towards the proximal end for the velocity difference between Region 2 and Region3, i.e., Stage II commences.

Thus, the governing equations for Stage II are

(23) $$\left\{ \begin{array}{l} \frac{dX_{2}}{dt}=-(v_{2}-v_{3}+c_{2})\\ \frac{dX_{3}}{dt}=v_{3}+c_{3} \end{array}\right.$$

(24) $$\left\{ \begin{array}{l} \frac{dv_{2}}{dt}=\frac{p(t)-\sigma_{0}(\rho \left(X_{2}\right))-\rho_{S}\rho \left(X_{2}\right)(v_{2}-v_{3})(v_{2}-v_{3}+c_{2})}{m_{2}+\rho_{S}\int_{0}^{X_{2}}\rho \left(x\right)dx}\\ \frac{dv_{3}}{dt}=\frac{\sigma_{0}(\rho \left(X_{2}\right))+\rho \left(X_{2}\right)(v_{2}-v_{3})(v_{2}-v_{3}+c_{2})-\sigma_{0}(\rho_{0})-\rho_{0}v_{3}(v_{3}+c_{3})}{m_{1}+\rho_{S}\int_{X_{2}}^{L_{2}}\rho \left(x\right)dx+\rho_{S}\rho_{0}(X_{3}-L_{2})} \end{array}\right.$$

The initial conditions of Stage II are *X*_1_ = *x*_11_, *X*_2_ = *x*_12_, *X*_3_ = *x*_13_, *v*_1_ = *v*_2_ = *v*_11_ = *v*_12_ and *v*_3_ = *v*_13_, where *x*_11_, *x*_12_, *x*_13_, *v*_11_, *v*_12_ and *v*_13_ are the Lagrangian coordinates of Shocks 1, 2 and 3, the velocities of Regions 1, 2 and 3 at the end of Stage I, respectively.

The velocity of Region 2 continues to decline until it satisfies the condition of *v*_2_ = *v*_3_ > 0, and then Shock 2 vanishes, i.e., Stage III commences. At this moment, Regions 1, 2, 3 and the cover plates *m*_1_ and *m*_2_ move as an entirety towards the distal end, as shown in Figure 4a. Similarly, the governing equations of the deformation at Stage III are

(25) $$\frac{dX_{3}}{dt}=v_{3}+c_{3}$$

(26) $$\frac{dv_{3}}{dt}=\frac{p(t)-\sigma_{0}(\rho_{0})-\rho_{S}\rho_{0}v_{3}(v_{3}+c_{3})}{m_{1}+m_{2}+2\rho_{S}\rho_{0}L_{2}/(2+\gamma )+\rho_{S}\rho_{0}(X_{3}-L_{2})}$$

The initial conditions are *X*_1_ = *x*_21_, *X*_2_ = *x*_22_, *X*_3_ = *x*_23_, *v*_1_ = *v*_2_ = *v*_3_ = *v*_21_ = *v*_22_ = *v*_23_, where *x*_21_, *x*_22_, *x*_23_, *v*_21_, *v*_22_ and *v*_23_ are the Lagrangian coordinates of Shocks 1, 2 and 3, the velocities of Regions 1, 2 and 3 at the end of Stage II, respectively. The governing equations of Stage I, Stage II and Stage III can also be solved by using the Runge-Kutta method. The stress distribution along the cladding at Stage I, II and III are also checked in Figure 4b, and the stresses at undeformed Regions do not exceed the local initial crushing stress.

## 4. Results and Discussion

### 4.1. Critical Length

The critical length, which defined as the minimum length of cellular sacrificial cladding to fully absorb the energy induced by the blast loading, is a vital target parameter to evaluate the cladding. However, the critical length of graded-uniform claddings cannot be directly obtained because it depends on the initial length *L*_2_ and the shock propagation behavior on the uniform layer within the end time. The end time *t*_m_ can be obtained by the law of momentum conservation that the impulse induced by the blast loading should equal to the impulse absorbed by the sacrificial cladding, i.e., *P*_0_*τ* = *σ*_0_*t*_m_. In order to obtain the critical length of graded-uniform sacrificial cladding, two constraints [20] are introduced to optimize the design of the thickness distribution of the PG-U and NG-U claddings.

Constraint I is that the proximal layer (graded cellular layer) is fully compacted exactly when the shock waves in this layer vanish. In detail, Shock 1 just reaches the end of the proximal layer when it vanishes for PG-U cladding, and Shock 2 just reaches the stop position of Shock 1 in the proximal layer when it vanishes for NG-U cladding. Applying this condition constrains, we can determine the optimal length of the proximal layer of graded-uniform cladding.

Constraint II is that the distal layer is fully compacted at the instant when the blast loading is fully absorbed. Thus, the thickness of the distal layer can be determined by the final stop position of the shock front in the uniform layer, i.e., Shock 2 for the PG-U cladding and Shock 3 for the NG-U cladding.

By considering Constraints I and II, the critical thickness distribution of graded-uniform cladding can be determined when the blast loading and cover plates are defined.

### 4.2. Propagation of Shock Wave in the Cladding

It is reported that the properties of cellular material can be denoted as functions of its relative density [41,42,43,44]. The two material parameters of R-PH model can also be expressed in power-law forms related to the relative density *ρ*, written as

(27) $$\left\{ \begin{array}{l} \sigma_{0}(\rho )=k_{1}\sigma_{S}\rho^{n_{1}}\\ C\left(\rho \right)=k_{2}\sigma_{S}\rho^{n_{2}} \end{array}\right.$$

where *σ*_S_ is the yield stress of the matrix materials, and *k*_1_, *k*_2_, *n*_1_, *n*_2_ are fitting parameters. Recently, Cai et al. [31] obtained the coefficients in Equation (27) by fitting the quasi-static compression curves of Voronoi honeycombs (as used in this paper to model the cellular materials) as *k*_1_ = 0.439, *k*_2_ = 0.127, and *n*_1_ = *n*_2_ =2. The solid material of Voronoi honeycombs for the present study is assumed to be elastic, perfectly plastic with Young’s modulus 66 GPa, Poisson’s ratio 0.3, yield stress 175 MPa and density *ρ*_s_ = 2700 kg/m^3^. Sacrificial claddings with specific density distribution are applied to demonstrate the propagation of shock waves in claddings for blast alleviation. Here, the mass per unit area of the cover plate is *m*_1_ = *m*_2_ = 2.7 kg/m^2^, the initial peak and decay time of the blast loading are *P*_0_ = 20 MPa and *τ* = 0.15 ms. The density of uniform cellular cladding *ρ*_0_ = 270 kg/m^3^, and the density-gradient parameter *γ* = 2/3 and −2/3 corresponding to PG-U and NG-U claddings, respectively. Thus, the critical thickness distribution of PG-U cladding can be determined as *L*_2_ = 91.6 mm and *L* = 272 mm; while the critical thickness of NG-U cladding as *L*_2_ = 140 mm and *L* = 298 mm.

**Figure 5:**
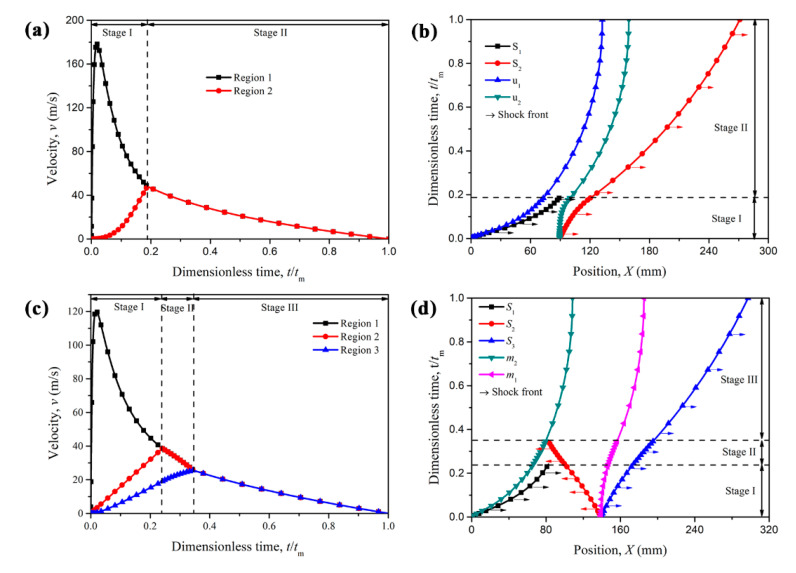
(**a**) Time histories of Region velocities and (**b**) the position of shock fronts for PG-U cladding (*γ* = 2/3, *L*_2_ = 91.6 mm and *L* = 272 mm); (**c**) time histories of Region velocities and (**d**) the position of shock fronts for NG-U cladding (*γ* = −2/3, *L*_2_ = 140 mm and *L* = 298 mm).

For the PG-U cladding, the time history of the velocities of Regions 1 and 2 (*v*_1_ and *v*_2_) and propagation behaviors of the shock fronts are depicted respectively in Figure 5a,b. At Stage I, the velocity of Region 1 (equals the velocity of cover plate *m*_2_), *v*_1_, first increases and then decreases. Meanwhile, the velocity of Region 2, *v*_2_, increases from 0 until it reaches the same speed of velocity *v*_1_. Then, Region 1 and Region 2 move together, and Stage II commences. From the shock propagation, see Figure 5b, Shock 1 just reaches the end of the proximal layer when Stage I comes to an end, which satisfies the condition of Constraint I. At Stage II, there exists only one shock wave, Shock 2, propagating to the support end (protected structure end). The cover plates *m*_1_ and *m*_2_ are moving with the same velocity *v*_2_, and the position difference of those two plates is the thickness of the compressed graded layer.

For the NG-U cladding, three shock waves are propagating simultaneously in the cladding once the blast loads. Similarly, the duration of Stage I is determined by the condition that the increasing velocity of Region 2 reaches the decreasing Region 1 velocity, i.e., *v*_2_ = *v*_1_. Then Shock 1 vanishes and Stage 2 begins. The end time of Stage II is determined by *v*_3_ = *v*_2_, which is also the commencement for Stage III. At Stage III, the part behind Shock 3 moves together, and its velocity is gradually reduced to zero due to the alleviation of the undeformed uniform layer, as shown in Figure 5c. The shock front propagation behavior of NG-U cladding is depicted in Figure 5d. Three shock fronts are described by the black, red and blue lines respectively, and they disappear at the end time of Stage I, Stage II and Stage III, respectively. The sum distance swept by Shocks 1 and 2 is just the thickness of graded layer *L*_2_, and this is precisely caused by Constraint I as mentioned above.

### 4.3. Parametric Analysis

**Figure 6:**
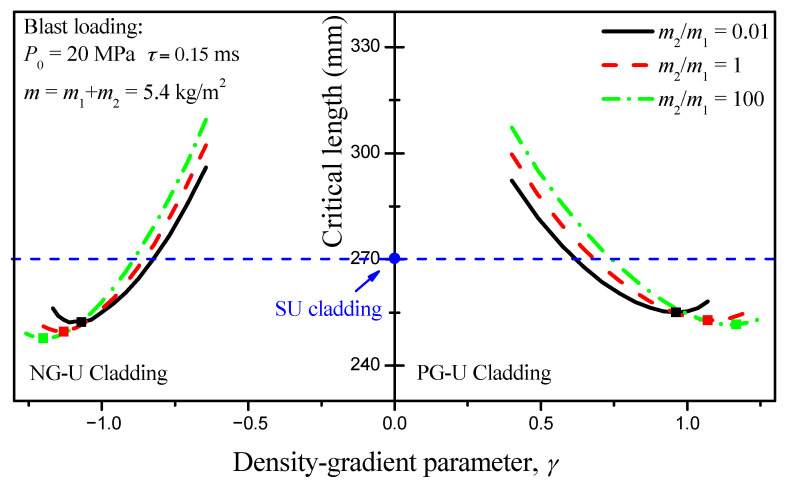
Variations of the critical length of the density graded-uniform cladding with gradient parameter *γ*.

The total mass and length of sacrificial cladding are two principal target indicators, which are associated with the cladding structural parameters, such as the attached mass distribution of cover plates and the density-gradient parameter *γ*. Considering the case that the total mass per unit area of the two cover plates in density graded-uniform claddings, i.e., *m* = *m*_1_+*m*_2_, is kept constant at *m* = 5.4 kg/m^2^, the critical length of the cladding decreases first and followed by a rise with the increasing of |*γ*|. By comparison with the uniform cellular materials cladding (called as “SU cladding” for short) [2], where the cover mass is equal to *m* and relative density of cellular materials is *ρ*_0_, the results indicate that the critical length of the density graded-uniform cladding is shorter than that of SU cladding within a certain range of |*γ*| variation, as shown in Figure 6. Furthermore, there exists a minimum length for each density graded-uniform cladding under a defined loading and cover mass. Notably, the changing trend of the critical length of density graded-uniform cladding with increasing of |*γ*| is not coincidental under different ratio of proximal cover plate mass to the middle one, *η* = *m*_2_/*m*_1_. From the perspective of the minimum critical length of cladding, the case of *η* = *m*_2_/*m*_1_ = 100 is more suitable for engineering design. In other words, the mass per unit area of the cover plate should all be concentrated on the proximal end, which has an advantage over the design of the critical length.

**Figure 7:**
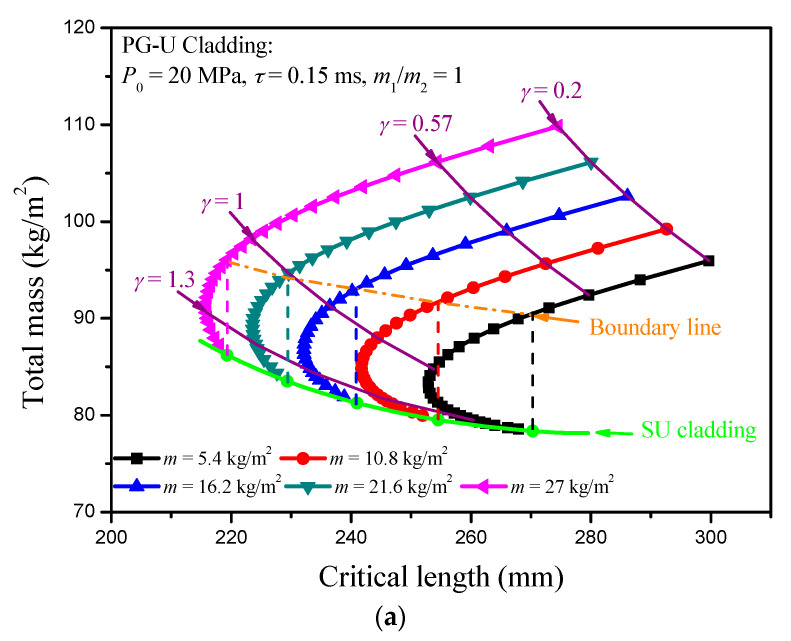

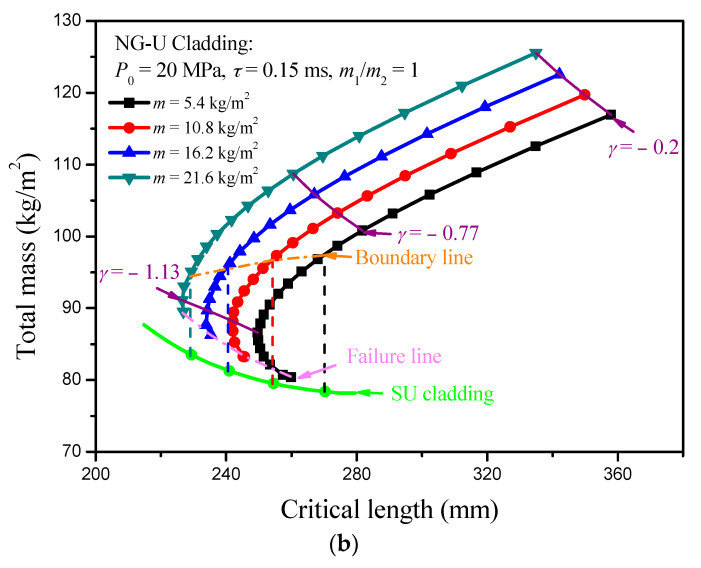
The total mass—critical length figure for (**a**) PG-U cladding and (**b**) NG-U cladding.

In fact, the blast loading can be treated as an impulse loading, so the mass per unit area of the cover plate indirectly decides the work done by the blast loading. Thus, a direct result can be deduced that the critical length of the sacrificial cladding decreases with increasing of the mass per unit area of cover plates, and it is verified by the numerical results, as shown in Figure 6. However, the total mass of the cladding presents an opposite law with the variation of the mass of cover plate. Under a fixed cover plate mass *m*, the critical length of PG-U cladding decreases first and then increases until it reaches the critical length value of SU cladding with the increasing of density-gradient parameter *γ*. At the same time, the total mass of PG-U cladding always shows a downward trend until it reaches the boundary of SU cladding. Therefore, the SU cladding is an excellent choice for the mass design indicator. In contrast, the PG-U cladding is more advantageous when choosing a large *γ* from the perspective of the critical length design indicator. There is a region in the total mass-critical length figure characterizing PG-U cladding should be preferred compared with SU cladding in critical length design. Two lines surround this region: one is indicated by SU cladding, the other is the boundary line where the critical length of PG-U cladding is equal to that of SU cladding. Similar results can be found for NG-U cladding. However, its variation curves of total mass and critical length cannot reach to the boundary of SU cladding with increasing of |*γ*|, and they are interrupted by the failure line which is determined by (*P*_0_ − *σ*(*ρ*(0)))/*m*_2_ = −4*γk*_1_*σ*_S_*ρ*_0_/(2+*γ*)*L*_2_*ρ*_S_^2^ as mentioned in Section 3.2. In general, *P*_0_ is larger than *σ*_0_(0), but this condition will be met when |*γ*| is large. Therefore, the advantageous region for NG-U cladding is surrounded by the boundary line and the failure line, as shown in Figure 7.

**Figure 8:**
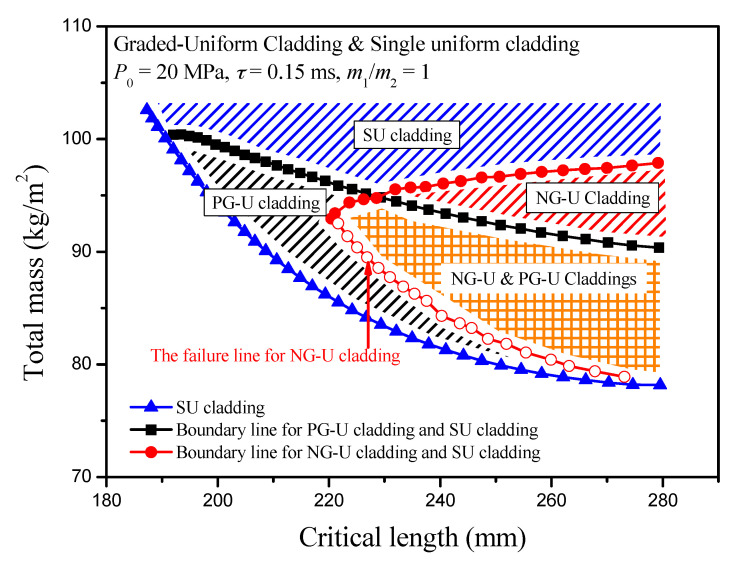
A partition diagram for the optimal length of sacrificial claddings.

Thus, partition diagrams in the total mass-critical length figure can be obtained when the minimum length of cladding is the design goal, where the blue, black and red shaded areas represent SU cladding, PG-U cladding and NG-U cladding, respectively, while the orange shaded area is the dual area for PG-U and NG-U claddings. Those shaded areas are distinguished by the lines representing SU cladding, the failure line for NG-U cladding, and two boundary lines as mentioned above. It is evident that SU, PG-U and NG-U claddings occupy different regions in the total mass-critical length figure, as shown in Figure 8, and it should identify different sacrificial claddings according to the actual demand. However, the SU cladding is a superior one compared with the PG-U and NG-U claddings, when total mass is the pursuit of goals.

## 5. Comparison with Cell-Based Finite Element Model

**Figure 9:**
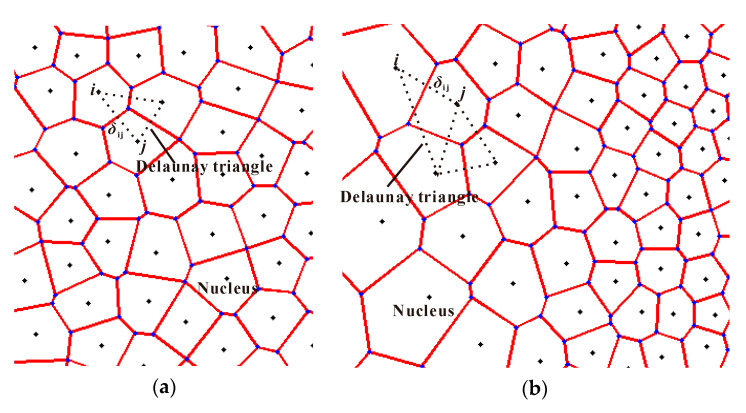
2D random Voronoi technique: (**a**) uniform honeycomb (k = 0.2); and (**b**) graded honeycomb (k = 0.2).

2D irregular honeycombs with a uniform cell-wall thickness are used and generated by utilizing the 2D Voronoi technique [45] in this study. The methodology of the Voronoi technique can generally be described in four main stages [22,46]. At first, *N* nuclei were randomly scattered in a given region. Secondly, the nuclei were imaged to the surrounding regions to make sure the boundary density is consistent with the preconcerted design. Thirdly, Delaunay triangulation is constructed, and then the Voronoi diagram is determined. Finally, the part of the Voronoi diagram of the given region is reserved for further analysis. It is reported that 2D graded cellular structures can be realized by switching the nuclei scatter strategy in the first Stage [22], as shown in Figure 9. The distance between any two nuclei *i* and *j* is required to be

(28) $$\delta_{\mathrm{ij}}\geq (1-k)\delta_{0}(\rho_{ij})=(1-k)\frac{2h}{\rho_{ij}}$$

where *k* is the cell irregularity, *δ*_0_(*ρ*) is the minimum distance between any two adjacent nuclei in a honeycomb structure with relative density *ρ*, *ρ*_ij_ is the local relative density at the middle point between nuclei *i* and *j*, and *h* is a presupposed cell-wall thickness. Thus, any 2D cellular structure with continuous density variation can be constructed according to the given local relative density distribution.

In this section, we consider the case that the blast loading is *P*_0_ = 30 MPa and *τ* = 0.15 ms, the mass per unit area of the cover plates are *m*_1_ = *m*_2_ = 2.7 kg/m^2^ and the density-gradient parameter is set as *γ* = ±1. Thus, the critical thickness distribution of PG-U cladding can be determined as *L*_2_ = 67.6 mm and *L* = 388.4 mm, while the critical thickness of NG-U cladding as *L*_2_ = 110 mm and *L* = 391.7 mm. In the cell-based FE simulations, a 2D graded Voronoi honeycomb structure (*γ* = 1) with an area of 67.6 × 100 mm^2^, a 2D uniform Voronoi honeycomb structure with an area of 320.8 × 100 mm^2^ and two rigid plates with an additional mass of 0.27 g respectively are assembled as an FE model for PG-U cladding. Similarly, a 2D graded Voronoi honeycomb structure (*γ* = −1) with an area of 110 × 100 mm^2^, a 2D uniform Voronoi honeycomb structure with an area of 281.7 × 100 mm^2^ and two rigid plates with an additional mass of 0.27 g respectively are assembled as an FE model for NG-U cladding. The length of specimens in the out-of-plane direction is 1 mm. ABAQUS/Explicit code was used to perform the FE simulations. Cell walls of specimens were modeled with S4R shell elements. The element size was set to be about 0.6 mm in-plane and 1 mm out-of-plane through a mesh sensitivity analysis. Thus, there are about 11,350 and 16,520 elements for the FE models of PG-U cladding and NG-U cladding, respectively. General contact with a slight friction coefficient of 0.02 is employed, as used in Zheng et al. [12]. To simulate an in-plane strain state, all the nodes were constrained in the out-of-plane direction. A pressure with exponential function (*P*_0_ = 30 MPa and *τ* = 0.15 ms), which can be directly called in ABAQUS/Explicit code, was used to simulate the blast load in the FE simulation.

**Figure 10:**
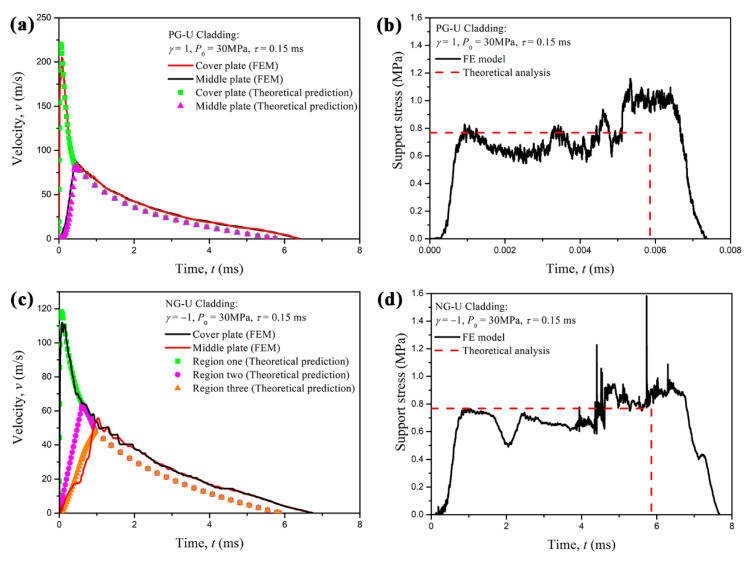
Comparison between cell-based FE results and theoretical predictions: (**a**,**b**) for PG-U cladding; (**c**,**d**) for NG-U cladding.

A comparison between the cell-based FE results and the theoretical predictions based on the R-PH shock model is presented in Figure 10. The velocity curves of the cover plate obtained from the analytical predictions and FE results are in good agreement, as shown in Figure 10a,c, which correspond to PG-U cladding and NG-U cladding, respectively. The cell-based FE results demonstrate that the proximal layer and the distal layer almost deform simultaneously once the claddings are applied to the blast load, and the middle plate velocities of cell-based FE results also conform with those of theoretical results very well, which can directly illustrate the reasonable of the assumption made in Section 3. The two layers also move together when the velocity of the proximal layer decreases to that of the distal layer for both graded uniform cellular sacrificial claddings.

The support stress is an important consideration, which is related to the effectiveness of the sacrificial cladding. The results indicate that the time history of the support stress obtained from the FE results is consistent with the theoretical solutions except it is slightly higher than the theoretical predictions in the later period, as depicted in Figure 10b,d. This tiny difference may be due to the action of an elastic reflected wave from the stationary support end and within the acceptable error. If only the proximal graded cellular layer is considered for sacrificial cladding, the maximum support stress will present a rising trend, which is disadvantageous for the anti-blast design of sacrificial cladding.

**Figure 11:**
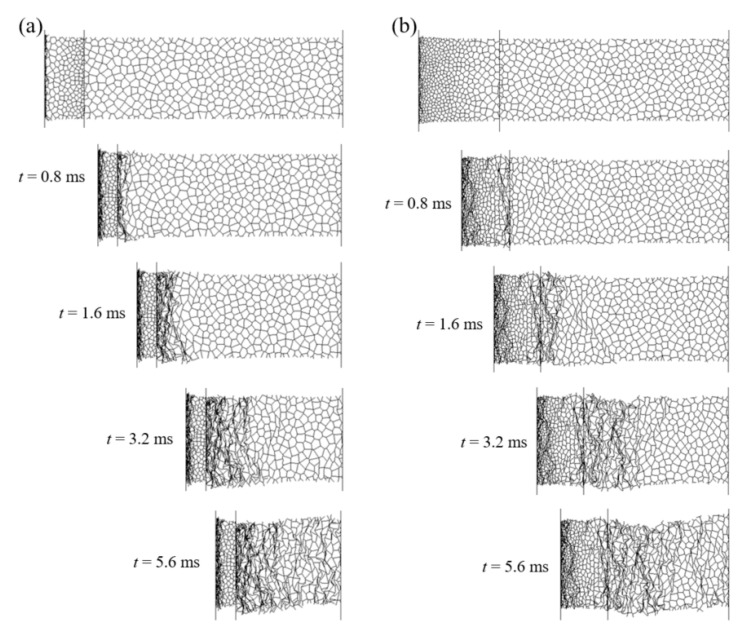
Deformation process of cellular sacrificial claddings obtained from the FE model: (**a**) PG-U cladding; (**b**) NG-U cladding.

Detailed deformation patterns of PG-U cladding and NG-U cladding under blast loading of *P*_0_ = 30 MPa and *τ* = 0.15 ms are shown in Figure 11a,b, respectively. It can be observed that two shock fronts initiate simultaneously in PG-U cladding once the blast loads, and three shock fronts commence simultaneously in NG-U cladding. Those phenomena are consistent with the assumptions of theoretical analysis. However, the crushing behavior presents random deformation bands at the later stage of compression for both claddings, because of the decrease of impact velocity. As the velocity of the attached mass becomes low at the later stage of compression, see Figure 10a,c, the deformation mode of the cellular sacrificial cladding changes into a transition or homogeneous mode [12,15,37,45], which can be verified by the random shear collapse bands in the uniform cellular layer. Thus, the results will be more accurate for cellular sacrificial cladding when considering both dynamic and quasi constitutive relations.

## 6. Conclusions

The dynamic responses of density graded-uniform sacrificial cellular claddings subjected to blast loading are investigated theoretically and numerically in this paper. Based on the rate-independent R-PH shock model of cellular materials and blast loading with an exponential attenuation function, differential equations governing the shock front propagation in density graded-uniform claddings were obtained and solved numerically with a fourth-order Runge-Kutta scheme.

Theoretical predictions reveal the characteristics of the motion law of cover plates and the shock front propagation in the claddings. There exist two shock fronts and two stages in the anti-blast response of the PG-U cladding, while there were three shock fronts and three stages in NG-U cladding. The influences of the cover plate mass and the density graded parameter *γ* on the critical length of sacrificial cladding were analyzed. The results illustrate that the cover plate mass distribution should concentrate on the proximal end, which is beneficial for the anti-blast design of both PG-U and NG-U claddings. Moreover, the optimal critical thickness of the graded-uniform cladding can be achieved by choosing a reasonable graded parameter when the mass ratio of the two cover plates is defined. Then, a critical length design partition diagram for SU, PG-U and NG-U claddings against the blast load is presented, and it illustrates that each cladding can contribute its talent according to the actual demand. The SU cladding is a superior choice compared with PG-U and NG-U claddings when pursuing the minimum total mass as the goal.

The numerical simulations of anti-blast behavior of sacrificial cladding are carried out by using the cell-based finite element model. The analytical predictions of graded-uniform claddings are compared with the cell-based FE results. Generally, good agreement is achieved and further confirms the correctness and effectiveness of theoretical analysis.
